# Fractures of the Lower Jaw

**Published:** 1859-02

**Authors:** O. C. Gibbs

**Affiliations:** Frewsburg, N. Y.


ARTICLE V.

### FRACTURES OF THE LOWER JAW.

BY O. C. GIBBS, M.D., FREWSBURG, N. Y.

EDITORS OF THE CHICAGO MEDICAL JOURNAL:

DEAR SIRS—In a recent number of your Journal, Dr. M. O. Heydock reports a fracture of the lower jaw, in which great difficulty was experienced in retaining the fractured parts in apposition. Dr. Heydock concludes his paper with the following remarks: "I believe the skull cap and straps here introduced will be found the best and simplest external appliance for all fractures of the jaw. It does not slip, like the roller, the pressure is distributed, and it can be loosened or tightened in a moment, with little or no trouble. It may not be a new idea, yet I do not remember ever having seen it mentioned."

The "skull cap and straps" are not altogether new; I have used them for years. In the February number of the *Buffalo Medical Journal*, for 1858, I reported a case of fracture of the inferior maxilla, which came under treatment in April, 1856, in which report the following language was used: "On examination, the inferior maxilla was found broken in *three* distinct places. The teeth in the upper jaw were entire, and the fragments of the bone were carefully moulded to an exact adaptation to the upper maxilla, with the design of making it subserve the purpose of an efficient splint to hold in place the fragments of the broken bone. In consequence of the great tumefaction, it was thought advisable to depart from the ordinary manner of applying bandages; it was thought that the pressure of the bandages upon the injured side of the face would, in consequence of its swollen condition, displace the fragments of the jaw inward. To prevent this, a splint somewhat of a horse-shoe shape, was fitted to the inferior surface of the fractured bone, projecting sufficiently beyond it on the sides to guard against lateral pressure. The splint was well padded and applied, *and held in place by suitable bandages, strapped to a* CAP OF FIRM CLOTH, *fitted to the head.*" This, in such cases, is a very desirable dressing, and with Dr. Heydock, we can say we believe it the "best and simplest external appliance for fractures of the jaw."

